# Performance and Cost-Effectiveness of Short Pitch-Based Carbon Fiber Reinforced Mortar Composite

**DOI:** 10.3390/ma14164693

**Published:** 2021-08-20

**Authors:** Md. Safiuddin, George Abdel-Sayed, Nataliya Hearn

**Affiliations:** 1 Angelo DelZotto School of Construction Management, George Brown College, 146 Kendal Avenue, Toronto, ON M5T 2T9, Canada; 2 Department of Civil Engineering, Faculty of Engineering and Architectural Science, Ryerson University, 350 Victoria Street, Toronto, ON M5B 2K3, Canada; 3 Department of Civil and Environmental Engineering, Faculty of Engineering, University of Windsor, 401 Sunset Avenue, Windsor, ON N9B 3P4, Canada; geosayed@yahoo.com (G.A.-S.); drhearn@gmail.com (N.H.)

**Keywords:** air content, carbon fibers, compressive strength, flexural strength, impact resistance, inverted slump cone flow, mortar, performance to cost ratio, unit weight, water absorption

## Abstract

This paper discusses the performance of the short pitch-based carbon fiber reinforced mortar (CFRM) composite considering its key properties and cost-effectiveness. Five different types of mortar composite were produced using 0–4% volume contents of short pitch-based carbon fibers. The mortar composites were tested for inverted slump cone flow (flow time and volume flow), unit weight, air content, compressive strength, flexural strength, impact resistance, and water absorption. The cost-effectiveness of CFRM was assessed based on the performance to cost ratio (PCR), which was calculated for each mortar composite, considering its workability, mechanical properties, and durability. The inverted slump cone volume flow was counted as a measure of workability, whereas the compressive strength, flexural strength, and impact resistance were considered as the major attributes of the mechanical behavior. In addition, the water absorption was used as a measure of durability. The test results revealed that the mortar composite made with 3% carbon fibers provided adequate workability, a relatively high unit weight and low air content, the highest compressive strength, excellent flexural strength, good impact resistance, and the lowest water absorption. It was also found that the PCR increased up to 3% carbon fibers. Beyond a 3% fiber content, the PCR significantly decreased. The overall research findings revealed that the mortar with 3% carbon fibers was the optimum and most cost-effective mortar composite.

## 1. Introduction

Cement-based materials typically exhibit brittleness and a low tensile strength, which can be improved by incorporating fibers [1,2,3]. Fibers are slender and elongated filaments in the forms of bundles, networks, or strands of any natural or manufactured material that can be distributed throughout the freshly mixed cement composites [4]. They are used in cement composites to improve their tensile, flexural, and impact behaviors with simultaneous increases in stiffness, toughness, and elongation at failure [5,6,7,8,9,10,11,12]. Fibers control the propagation of cracks under loading, and thus enhance the tensile strength, flexural strength, and impact resistance of cement composites [2,13,14,15,16]. Fiber reinforced cement composite can continue to carry a considerable amount of loading after the occurrence of cracking [17]. This means that the low fracture toughness of plain cement composite is rectified significantly in the presence of fibers. Consequently, a more stable crack growth is induced before the cracks become unstable. Therefore, the cracks can also sustain a much wider opening before instability occurs [18]. In fact, the fibers act as stress-transfer bridges [8,14] and thereby reduce the stress intensity at the crack tips.

The most common fibers used in mortar and concrete are steel, glass, and polymeric fibers such as polypropylene and polyvinyl alcohol, as well as advanced fibers like aramid and carbon fibers [8,9,10,11,12,13,18]. The Japanese started the use of short carbon fibers in cement composites in a form known as carbon fiber reinforced composite (CFRC). CFRC is advantageous over polypropylene, glass, and steel fiber reinforced cement composites regarding finishability, weatherability, mixability, thermal resistance, and long-term chemical stability in aggressive environments [19]. Mostly short pitch-based carbon fibers have been used in CFRC. Pitch-based carbon fiber appeared in Japan in 1980 [20] and became prominent owing to its lower cost than other advanced fibers.

In the past, significant research was carried out to investigate the potential of pitch-based carbon fibers [16,21,22,23,24,25,26,27,28,29]. In particular, the mass production and industrialization of CFRCs went on in Japan in the form of curtain walls for high-rise buildings [20,21,28,29]. Carbon fibers were first used at a large scale in the form of lightweight cladding tile panels for the Al-Shaheed Monument in Iraq in 1982 [21]. After this successful application, CFRC was used structurally in the exterior curtain wall of the 37-storey Ark-Mori Building in Japan in 1986 [21]. Then, CFRCs were applied successfully in Japan to construct the curtain walls of Suidobashi Building of the Tokyo Dental College, Shinagawa-Ku Building, Hitachi Civic Center, Nihonbashi-Honcho Building, Sea Fort Square, Edo-Tokyo Museum, and the United Nations University [25,30].

The invention of pitch-based carbon fibers indeed allowed for a breakthrough in the market of advanced composite materials. Pitch-based carbon fiber was accepted as an excellent reinforcement for cementitious composites, and was used extensively for many applications, as depicted above, due to its low price compared with other advanced fibers [19,21,25,29]. Yet, the cost of pitch-based carbon fiber is higher than that of the other constituents of cement composites. Hence, the cost-effectiveness of CFRC should be determined before any commercial applications. However, limited studies have been conducted considering a cost analysis of CFRC in relation to its performance regarding workability, physical and mechanical properties, and durability.

The properties of fiber reinforced cement composites vary with the volume content of fibers. Shah and Rangan [31] showed that the higher volume content of fibers increased the tensile strength and toughness of the composites almost linearly. The higher fiber volume content also consistently increased the ductility and energy absorption capacity of the cement composites [32,33]. However, the higher fiber volume content was seen to significantly affect the mixing and placement of the fiber composites [18,34]. On the other hand, fiber reinforced cement composites exhibited a brittle post-peak failure pattern at a lower fiber volume content in the presence of silica fume [18]. This is because the presence of silica fume decreases the fracture toughness and makes the material more brittle [35,36]. Therefore, a higher fiber volume content needs to be used in the presence of silica fume. On the contrary, it is economical to keep the fiber volume content as low as possible for many fibers because of their higher price. Therefore, the optimization of the fiber content in cement composites is required. Limited studies have focused on this aspect of fiber reinforced cement composites, particularly CFRC.

This study examines the performance of the carbon fiber reinforced mortar (CFRM) composite with respect to its workability, mechanical properties, and durability. The effects of different volume contents of carbon fibers on these properties were investigated. Most importantly, a cost-performance analysis of different CFRM composites was carried out to determine the most cost-effective mortar composite.

## 2. Research Significance

CFRM composite is advantageous over conventional mortar composite due to its improved mechanical properties, particularly its flexural strength and impact resistance. However, the higher price of carbon fibers compared with other ingredients increases the material cost of the CFRM composite. The present study shows that the increased material cost of the CFRM composite can be compensated by its significantly improved performance. In this study, the workability (flow time and volume flow), mechanical properties (compressive and flexural strengths and impact resistance), and water absorption (a measure of durability) of different mortar composite mixes were determined. These properties are vital for a CFRM composite if it is intended for use in infrastructure (e.g., buildings, bridges, and pavements). In the present study, the effects of short pitch-based carbon fibers on the above-mentioned properties of mortar composite were investigated, and the optimum fiber content was determined based on both performance and cost. The research findings revealed that the optimum carbon fiber content was 3%, which made the CFRM composite the most cost-effective by providing the highest performance to cost ratio. The maximum improvement in water absorption and compressive strength occurred for CFRM3 made with 3% carbon fibers. Among all of the CFRMs, CFRM3 had the lowest volume of entrapped air voids and the highest unit weight. In addition, CFRM3 possessed excellent flexural strength and good impact resistance. Therefore, the optimum carbon fiber content leading to the highest performance to cost ratio of the CFRM composite was 3%. It is expected that such an outcome from this study will guide the construction industry to produce cost-effective CFRM composite for multiple applications in infrastructure.

## 3. Materials and Methods

### 3.1. Constituent Materials

Natural river sand, ordinary Portland cement, silica fume, short pitch-based carbon fibers, normal tap water, and naphthalene-based superplasticizer were used to produce the mortar composites. The maximum particle size of the sand was 2.36 mm. The cement conformed with the standard specification for ASTM Type I Portland cement given in ASTM C150/C150M–16 [37]. Silica fume conformed with the ASTM standard specification given in ASTM C1240–15 [38]. Short pitch-based carbon fibers were used in the form of multi-filament strands—the length and dimeter of filament was 10 mm and 17 μm, respectively. Tap water complied with the requirements, as specified in ASTM C94/C94M–16 [39]. Superplasticizer conformed with the specification of Type F high-range water reducing admixture given in ASTM C494/C494M–16 [40]. The key properties of the constituent materials are given in Table 1.

### 3.2. Mix Proportions of Various Mortar Composites

In total, five different types of mortar composite mixes, including a control mix, were designed with a water-binder ratio of 0.35 and a sand-binder ratio of 0.50. The control mix was ordinary Portland cement mortar (OPCM). Four carbon fiber reinforced mortars, namely CFRM1, CFRM2, CFRM3, and CFRM4, were designed with a fiber content of 1%, 2%, 3%, and 4% by mortar volume, respectively. For all of the above mortar mixes, silica fume was considered as a 15% replacement of cement by weight. In addition, while calculating the mix proportions of the different mortar mixes based on the absolute volume method, a 1% air content was considered for OPCM, whereas a 4% air content was assumed for CFRM1. The assumed air contents were 5%, 6%, and 7% for CFRM2, CFRM3, and CFRM4, respectively.

The mix proportions for all of the mortar composite mixes were finalized based on their workability performance. A maximum inverted slump cone flow time of 30 s was used as the acceptability criterion for a mortar composite mix to be workable for casting specimens under vibration. This flow time was decided based on the findings from the research of Johnston [41]. The dosage of the superplasticizer by weight of the binder (cement plus silica fume) was varied to get the flow time of the mortar mixes well below 30 s. The finalized mix proportions of the different mortar composite mixes per unit volume (1 m^3^) are shown in Table 2. Before batching, the proportions of sand and water were adjusted for each mortar mix, considering the water absorption of sand as well as the water contribution of the superplasticizer.

### 3.3. Preparation and Testing of Fresh Mortar Composites

The mortar composites were prepared based on the mix proportions shown in Table 2. A pan-type mixer with a capacity of 0.1 m^3^ was used for preparing the fresh mortar composites. Carbon fibers were hand-sprinkled in the running mixer and mixed for 3 min with the other constituent materials. The total mixing time was 6 min for CFRM1, CFRM2, CFRM3, and CFRM4. In contrast, the total mixing time was 4 min for the OPCM. The details of the mix procedure are given in [42].

The freshly mixed mortars were sampled for testing the inverted slump cone flow (flow time and volume flow), unit weight, and air content. The sampling was conducted in accordance with ASTM standard practice [43].

#### 3.3.1. Inverted Slump Cone Test

The inverted slump cone test was conducted according to ASTM C995 [44], with some exceptions for the shape of the container housing the cone and the filling of the cone with highly fluid OPCM. In the present study, a square wooden container was used to ease the insertion of a metal plate just below the lower end of the inverted slump cone. In addition, no vibrator was used in the case of OPCM while measuring its flow time because this mortar mix was self-flowing.

#### 3.3.2. Unit Weight and Air Content Test

The unit weight and air content of the freshly mixed mortar composites were determined according to the test procedures given in ASTM C138/C138M [45], with some exceptions for OPCM. In the case of OPCM, the cylindrical measure was filled in one layer without any vibration, as this mortar mix was self-compacting.

### 3.4. Preparation and Testing of Hardened Mortar Composites

The freshly mixed mortar mixes were used to prepare the test specimens for determining the mechanical properties of the mortar composites. For the flexure test, the beam specimens with dimensions of 400 mm (length) × 100 mm (height) × 75 mm (width) were cast in reusable metal molds. The Ø100 mm × 200 mm cylinder specimens for the compression test were fabricated using single-use plastic molds. For the impact test, the Ø150 mm × 62.5 mm cylinder specimens were obtained by cutting the Ø150 mm × 300 mm cylinders cast in reusable metal molds. On the other hand, the 50 mm × 50 mm × 50 mm cubes for the water absorption test were processed from the 150 mm × 150 mm × 150 mm cubes, which were also cast in reusable metal molds. ASTM standard practice [46] was followed for molding the test specimens in the laboratory. In the cases of the four CFRMs, the mortar mix was compacted by vibration while casting the test specimens. In contrast, no vibration was used for the test specimens made of OPCM.

The test specimens were de-molded after 24 h. The bug holes, if any, were filled by neat cement paste. Then, the specimens were marked and transferred to the water tank for curing. The specimens were water-cured until the time of testing at 28 days. The curing temperature was 23 ± 2 °C. ASTM standard practice [46] was followed when curing the specimens, except for using lime in the water.

#### 3.4.1. Compression Test

The compressive strength of the mortar composites at 28 days was determined according to ASTM C39/C39M [47]. Three Ø100 mm × 200 mm cylinder specimens were used in this test. Before testing, they were capped with sulfur mortar following the standard practice given in ASTM C617/C617M–15 [48]. During testing, the ultimate load was recorded from the dial gauge of the compression testing machine and was used to calculate the compressive strength of the mortar composite. The average compressive strength was determined based on the test results of the triplicate Ø100 mm × 200 mm cylinder specimens.

#### 3.4.2. Flexure Test

The flexural strength of the mortar composites at 28 days was determined according to ASTM C 1018-97 [49]. Three 400 mm (length) × 100 mm (height) × 75 mm (width) beam specimens were used in this test. The loading was applied continuously without any shock by means of a hydraulic jack. The loading arrangement was connected to the data acquisition system. The ultimate load was found from the gathered data and was used to calculate the flexural strength of the mortar composite. The average flexural strength was determined from the test results of the triplicate 400 mm (length) × 100 mm (height) × 75 mm (width) beam specimens.

#### 3.4.3. Impact Test

The impact resistance of the mortar composites at 28 days was determined in accordance with the test method recommended by ACI Committee 544 [50]. The amount of impact energy required to cause an opening of the cracks until failure was determined using this test method. The test set-up consisted of a standard 4.5 kg (10 lb) compaction hammer with a 457 mm (18 in) drop conforming with the ASTM specification for compacting hammer for use in a laboratory [51], a 63.5 mm (2.5 in) diameter steel ball, and a steel positioning fixture to hold the cylinder specimen. The test was performed using triplicate Ø150 mm × 62.5 mm cylinder specimens, which were prepared by cutting a Ø150 mm × 300 mm cylinder. The impact test was conducted by dropping the hammer repeatedly on the steel ball supported by the specimen, while observing the formation of the cracks and failure of the specimen. The number of blows required for the failure of the specimen was determined and used to compute the impact resistance. The average impact resistance was obtained from the test results of the triplicate Ø150 mm × 62.5 mm cylinder specimens.

#### 3.4.4. Water Absorption Test

The water absorption of the mortar composites at 28 days was determined according to the cold-water method, as specified in ASTM C1195-03 [52]. Three 50 mm × 50 mm × 50 mm cube specimens were used in this test. The amount of water absorbed over a period of 48 h was measured so as to calculate the water absorption of each cube specimen. The average water absorption was determined from the test results of the triplicate cube specimens.

### 3.5. Calculation of Cost-Effectiveness of CFRM

Carbon fibers are costlier than any other material components of CFRM. Therefore, the cost-performance optimization of the CFRM mix is desirable to make it cost-effective. The performance and cost of CFRM were taken into consideration to evaluate its cost-effectiveness. The idea introduced by Nehdi et al. [53] was used to examine the cost-effectiveness of CFRMs and to obtain an optimal mix, considering their performance with respect to workability, mechanical properties, and durability. The volume flow as a measure of workability, the compressive strength, flexural strength, and impact resistance as the measures of mechanical performance, and the water absorption as a measure of durability of the mortar composites were taken into consideration to determine the cost-effectiveness in relation to the overall performance. As a basis for comparison between CFRMs and OPCM regarding the performance and cost-effectiveness, a parameter called performance to cost ratio (PCR) was determined for each mortar using the following equation.

(1) $$PCR=\frac{V_{ff}+\left(C_{sf}\times F_{sf}\times I_{rf}\times W_{arf}\right)}{C_{f}}$$

where,

*C_f_* = Cost factor
*V_ff_* = Volume flow factor
*C_sf_* = Compressive strength factor
*F_sf_* = Flexural strength factor
*I_rf_* = Impact resistance factor
*W_arf_* = Water absorption resistance factor

The values of *C_f_*, *V_ff_*, *C_sf_*, *F_sf_*, *I_rf_*, and *W_arf_* for the CFRMs can be determined after dividing their unit cost, volume flow, compressive strength, flexural strength, impact resistance, and water absorption by the corresponding unit cost and property of OPCM. Considering this computation process, Equation (1) can be modified as follows:

(2) $$PCR=\frac{V_{fm}/V_{fc}+\left(C_{sm}/C_{sc}\times F_{sm}/F_{sc}\times I_{rm}/I_{rc}\times W_{ac}/W_{am}\right)}{C_{m}/C_{c}}$$

where,

*C_c_* = Cost of 1 m^3^ of the control mortar
*C_m_* = Cost of 1 m^3^ of any mortar
*V_fc_* = Volume flow factor of the control mortar
*V_fm_* = Volume flow factor of any mortar
*C_sc_* = 28-day compressive strength of the control mortar
*C_sm_* = 28-day compressive strength of any mortar
*F_sc_* = 28-day flexural strength of the control mortar
*F_sm_* = 28-day flexural strength of any mortar
*I_rc_* = 28-day impact resistance of the control mortar
*I_rm_* = 28-day impact resistance of any mortar
*W_ac_* = 28-day water absorption of the control mortar
*W_am_* = 28-day water absorption of any mortar

The PCRs were determined for all of the mortar composites using Equation (2), and their cost-effectiveness was evaluated from the obtained PCRs. The higher the PCR, the more cost-effective the mortar with respect to its workability, mechanical properties, and durability. Eventually, the optimum mix was defined based on the properties and PCRs of various CFRMs.

## 4. Test Results and Discussion

### 4.1. Properties of Fresh Mortar Composites

The properties of the different freshly mixed mortar composites are shown in Table 3. The flow time and volume flow of the freshly mixed mortar composites were examined to measure their workability. The flow time was recorded directly during the inverted slump cone flow test, whereas the volume flow was calculated based on the volume of mortar composite in the inverted slump cone and its flow time. The air content of each mortar composite was determined from the same test used for measuring its unit weight.

#### 4.1.1. Inverted Slump Cone Flow

The flow time of mortar composite increased with the increase in the carbon fiber volume content (refer to Table 3). Therefore, the volume flow of CFRM deceased significantly as the fiber content increased. This implies that the workability of CFRM declined substantially with the increased amount of carbon fibers. Similar trends were observed by Park et al. [16], Akihama et al. [20], Bayasi and Soroushian [54], and Banthia et al. [55]. In this study, the flow time varied from 4 s to 16.5 s. Generally, a flow time in the range of 8–30 s infers that the fresh composite is appropriate for casting with vibration [41]. Thus, the mortar composites including 1–4% carbon fibers possessed sufficient workability for placing in molds or forms to make hardened elements.

The overall inverted slump cone flow results (see Table 3) show that the higher carbon fiber volume content increased the flow time, thus indicating a lower workability of the CFRM composite. CFRM4, which included 4% carbon fibers, had a lower workability compared with CFRM1 and CFRM2, which had 1% and 2% carbon fibers, respectively. This is because the higher surface area of the carbon fibers tends to restrain the mobility of the fresh mortar composite [42,56] due to increased water demand to entirely wet the constituent materials. Therefore, a comparatively high superplasticizer dosage was required to make CFRM4 workable. CFRM1 (1% carbon fibers) and CFRM2 (2% carbon fibers) required a 2% and 3% superplasticizer dosage, respectively. In comparison, CFRM3 (3% carbon fibers) and CFRM4 (4% carbon fibers) needed a 4% and 5% superplasticizer dosage, respectively. In this context, it should be mentioned that there was no visible fiber distribution problem and no fiber balling appeared during the mixing process when the mortar composite mixes were produced using the selected superplasticizer dosages.

#### 4.1.2. Unit Weight and Air Content 

The unit weight of CFRM decreased in the presence of carbon fibers, as is obvious from Table 3. Park et al. [16] and Akihama et al. [20] obtained similar results from their studies. The decline in the unit weight of the CFRM composite occurred due to lightweight carbon fibers and increased air content. Among all of the solid constituents of CFRM, short pitch-based carbon fibers had the lowest specific gravity (refer to Table 1). In addition, the incorporation of carbon fibers increased the volume of entrapped air voids in the mortar composite by decreasing its workability. These two factors mainly contributed to decrease the unit weight of the CFRM composite. However, the decrease in unit weight was the lowest for CFRM3, which included 3% carbon fibers. This is because CFRM3 had the lowest air content among all of the CFRMs (refer to Table 3). This indicates that the constituent materials became well-packed in CFRM3 when it was compacted under vibration, as compared with the other CFRMs.

The air content of the CFRM composites was significantly higher than that of OPCM. Furthermore, the air content increased to a greater extent with the higher volume content of carbon fibers, as evident from Table 3. This is because the presence of carbon fibers in the mortar impedes the movement of air voids to the surface during casting and compaction, thus entrapping them in the fresh composite [18,42]. However, it is evident from Table 3 that CFRM3 had the lowest air content. It implies that relatively less air voids were entrapped in the CFRM composite when 3% carbon fibers were used. This is for the same reason as explained above in the case of unit weight.

### 4.2. Properties of Hardened Mortar Composites

The hardened mortar composites were tested for their compressive strength, flexural strength, impact resistance, and water absorption at 28 days. The average results of the different mortar composites obtained from the triplicate specimens are given in Table 4.

#### 4.2.1. Compressive Strength

**Figure 1:**
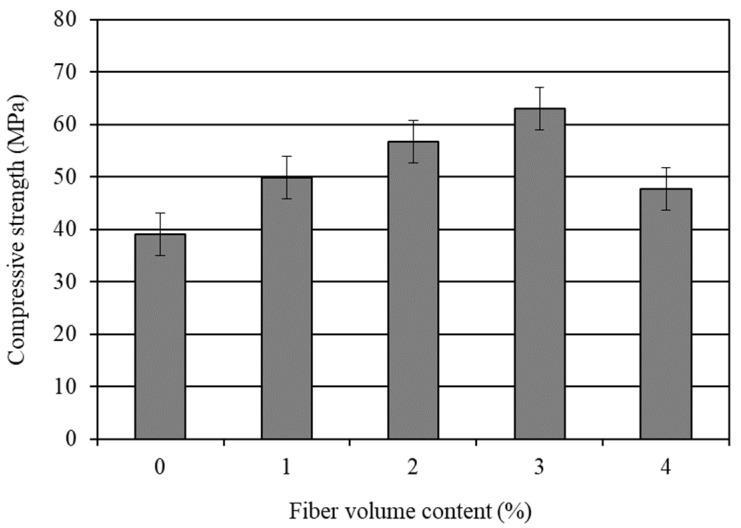
Influence of carbon fibers on the 28-day compressive strength of mortar composite.

The average 28-day compressive strength results of the water cured mortar composites are presented in Table 4. The influence of different volume contents of the carbon fibers on the compressive strength of CFRM is graphically shown in Figure 1. The standard error associated with the compressive strength results is also given in this figure. It is obvious from Figure 1 that the compressive strength of the CFRM composite increased with the inclusion of fibers for up to a 3% volume content, and then decreased sharply. Ohama et al. [29] observed a similar effect of the carbon fiber volume content on the compressive strength of cement composites. The compressive strength also dropped in their study beyond the 3% fiber content. However, it should be mentioned that they produced carbon fiber reinforced cement paste, not mortar.

The influence of the fiber content is not always beneficial for the compressive strength of the CFRM composite [57,58]. It greatly depends on the workability, air content, and extent of compaction of the mortar composite [18,56]. Although the fibers increase the compressive strength, it is rather low. This is because the fibers cannot provide a good reinforcement in compression compared with their superior reinforcing effect under tension. It is evident from Table 4 that the maximum increase in compressive strength (61%) after 28 days occurred for CFRM3 made with 3% carbon fibers. Similar findings were reported by several other researchers who observed that carbon fibers could enhance the compressive strength of cement composites [59,60,61,62]. The unit weight and air content results of CFRM3 (refer to Table 3) support its maximum increase in compressive strength obtained in the present study. The increase in unit weight and the decrease in air content usually improve the compressive strength of the mortar composite [56]. CFRM3 including 3% carbon fibers possessed a comparatively high unit weight but low air content. Furthermore, the carbon fibers increase the interfacial bond strength in the composite material [60,63]. This is due to their inherent surface roughness. In addition, the attachment of finer cement and silica fume particles onto the carbon fibers enhances their surface roughness [18]. In the present study, the workability of CFRM3 was relatively good, as indicated by its flow time of 8.5 s, which is much lower than the maximum acceptable limit of 30 s. Therefore, CFRM3 was compacted well under vibration, which is evident from its unit weight and air content results. In such conditions, the interfacial bond strength can also be increased. As a result, CFRM3 provided the highest compressive strength. On the contrary, CFRM4 made with 4% carbon fibers had the lowest compressive strength among the four CFRMs (refer to Table 4). The main reason is that it was not well-compacted because of its relatively low workability. Therefore, an excessive amount of air bubbles remained entrapped in the mix, which had a negative impact on the compressive strength of CFRM4. Thus, carbon fibers can improve the compressive strength of the mortar composite if used at a volume content without affecting the workability of the mortar mix. In addition, the decrease in compressive strength could be due to the retarding effect of the excessive superplasticizer, as observed by Pigeon et al. [64]. As a relatively high dosage of superplasticizer was used in CFRM4, it might retard the setting of cement, and thus could have a negative impact on its compressive strength. Such a retarding effect is not expected for the carbon fibers at any volume content, because they are a chemically inert material and therefore are not anticipated to interfere with the hydration reactions of the binder materials.

#### 4.2.2. Flexural Strength

**Figure 2:**
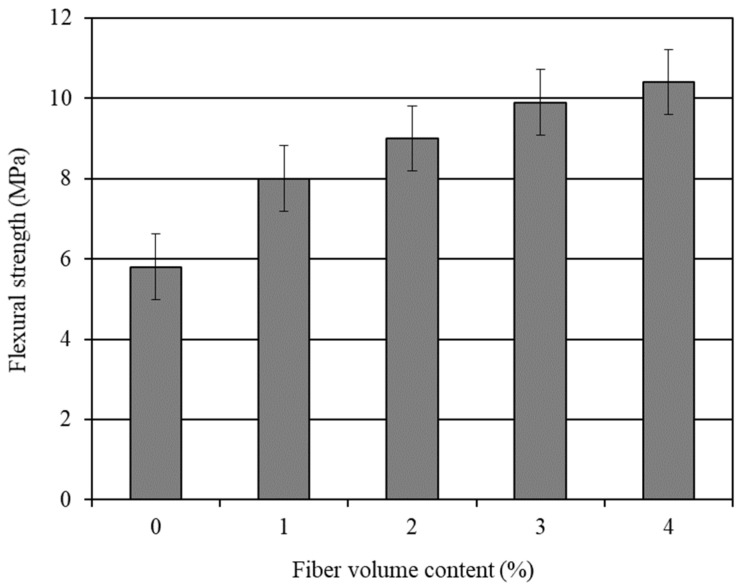
Influence of carbon fibers on the 28-day flexural strength of mortar composite.

The average 28-day flexural strength results of the various mortar composites are shown in Table 4. The effect of the carbon fiber content on the flexural strength of the mortar composite is graphically presented in Figure 2. The standard error associated with the flexural strength results is also shown in this figure. It is obvious from Figure 2 that the flexural strength of the CFRM composite increased with the increase in the carbon fiber content. Akihama et al. [20], Banthia and Sheng [23], and Kim and Park [65] observed a similar trend for the flexural strength of CFRC.

The incorporation of carbon fibers significantly increased the flexural strength of the mortar composite. The mortar containing a carbon fiber volume content of 4% (CFRM4) provided the maximum flexural strength. It provided 78% more flexural strength than OPCM. On the other hand, the mortar with 3% carbon fibers offered 70% more flexural strength than OPCM. The increase in flexural strength with the inclusion of carbon fibers is primarily because of their better reinforcing and bonding effects, resulting from the improved densification of the microstructure in the presence of silica fume and a superplasticizer [18,56]. Moreover, the orientation of the carbon fibers in the beam specimens was conducive to increase the flexural strength of the mortar composite. Sakai et al. [66] stated that carbon fibers are likely to be oriented at a right angle to the oscillating waves of a vibrator. This indicates that the compaction by a vibrator caused a 2D fiber orientation parallel to the axis of the beam specimens, which were used in the flexure test. Therefore, the fibers seemingly became oriented along the direction of the major tensile stress and thereby contributed to the greater flexural strength.

#### 4.2.3. Impact Resistance

**Figure 3:**
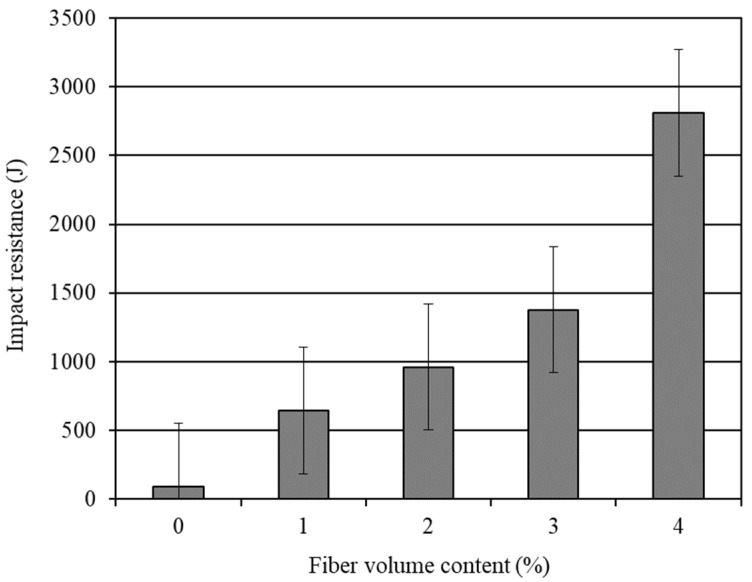
Influence of carbon fibers on the 28-day impact resistance of mortar composite.

The average 28-day impact resistance of the different water cured mortar composites is shown in Table 4. The effect of the carbon fiber volume content is graphically presented in Figure 3. The standard error associated with the impact resistance results is also shown in this figure. It is obvious from Figure 3 that the impact resistance increased markedly with the increased carbon fiber content. The mortar composite with 4% carbon fibers (CFRM4) offered a maximum impact resistance. The impact resistance of CFRM4 was 29.86 times that of OPCM. On the other hand, the impact resistance of the mortar composite with 3% carbon fibers (CFRM3) was 14.64 times that of OPCM. Limited studies have been conducted to examine the impact resistance of the CFRM composite using the ACI drop-weight test. Ohama et al. [29] investigated the impact resistance of CFRC operating a similar device and obtained comparable results. In addition, Soroushian et al. [10] carried out the ACI drop-weight test on a CFRC containing lightweight aggregates and observed an enormous enhancement in the impact resistance with the increased fiber content, similar to the improvement obtained in the present study. The superior improvement in the impact resistance of CFRM composite is chiefly attributed to the increased anchorage of the carbon fibers and the enhanced interfacial bond between fibers and matrix in the presence of silica fume.

#### 4.2.4. Water Absorption

**Figure 4:**
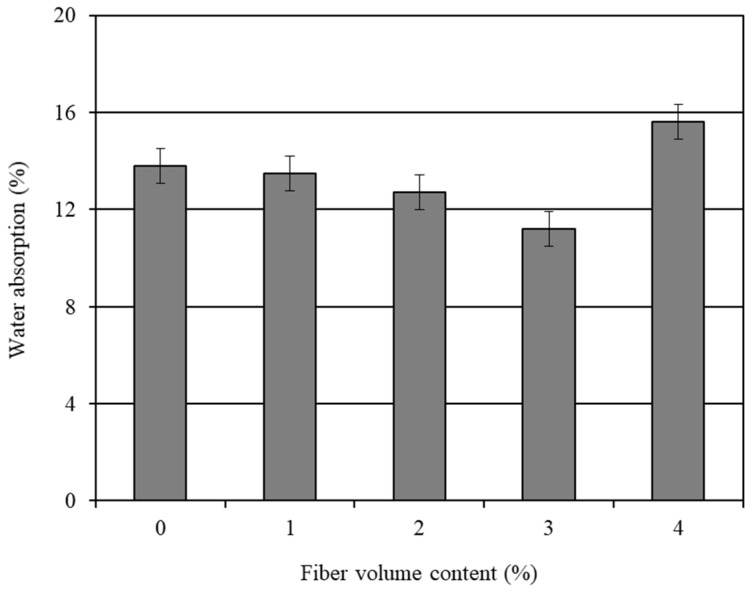
Influence of carbon fibers on the 28-day water absorption of mortar composite.

The average 28-day water absorption results of the various mortar composites are shown in Table 4. The influence of the carbon fiber volume content on the water absorption is graphically presented in Figure 4. The standard error associated with the water absorption results is also shown in this figure. It is obvious from Figure 4 that the water absorption decreased up to a 3% volume content of the carbon fibers, and then increased with the 4% fiber content. Limited studies were conducted to assess the water absorption of the CFRM composite. Ohama et al. [29] executed the water absorption test on CFRC and found that the water absorption decreased with the increase in the carbon fiber volume content up to 5%. A similar trend of reduction in water absorption was observed in this study for the carbon fiber volume content varying in the range of 1–3%.

The mortar composite CFRM3, which included 3% carbon fibers, had a 18.62% lower water absorption than OPCM. In comparison, the mortar composite CFRM4, made with 4% carbon fibers, had a 12.75% higher water absorption than OPCM. This is due to the increased capillary pores and air voids of CFRM4, as understood from its unit weight and air content results. Particularly, the capillary porosity largely influenced the water absorption property of CFRM4. The higher the capillary porosity, the greater the water absorption [56,67]. The unit weight and air content results of CFRM3 indicate that it was well-compacted due to its sufficient workability at an adequate dosage of superplasticizer. Therefore, it is likely that CFRM3 had a lower capillary porosity. In addition, the volume of entrapped air voids was relatively low in this mortar composite. As a result, CFRM3 exhibited the lowest water absorption.

The use of a superplasticizer and silica fume was conducive to decrease the water absorption of the CFRM composite through reduced porosity. A superplasticizer allows for a substantial reduction in the amount of mix water and thereby decreases the capillary porosity of the mortar composite [18,56]. Moreover, silica fume contributes to decreasing the capillary porosity of cement composites [68,69] through its micro-filling ability and pozzolanic activity. In addition, it improves the interfaces of sand particles and carbon fibers [70,71] due to pore filling. However, all these improvements greatly depend on the workability of CFRM. A high workability enhances the degree of compaction and thus plays an important role in reducing the capillary pores and entrapped air voids in a mortar composite [18,42]. Excessive capillary pores were likely to exist in CFRM4 because it was too stiff due to its low workability, despite using a relatively high dosage of superplasticizer and the same amount (15%) of silica fume. Moreover, this mortar composite contained the highest amount of entrapped air voids (refer to Table 3) because it had the lowest workability. Therefore, CFRM4 had the maximum water absorption.

## 5. Cost-Effectiveness of CFRM

The cost-effectiveness of CFRM was assessed based on the PCRs (performance to cost ratios) of different mortar composites. The PCRs were calculated based on Equation (2) comparing the performance of mortar composites with respect to the workability, mechanical behavior, and durability per unit cost. The volume flow was used as a measure of workability, whereas the compressive strength, flexural strength, and impact resistance were used to assess the mechanical behavior. Furthermore, the water absorption was used as a measure of durability. The unit cost was determined from the recent prices of the constituent materials of the mortar composites. They were gathered from the manufacturers and suppliers, except for the water. The price of tap water was obtained from the city’s water rates. The summary of calculations for the PCRs of different CFRMs is given in Table 5.

**Figure 5:**
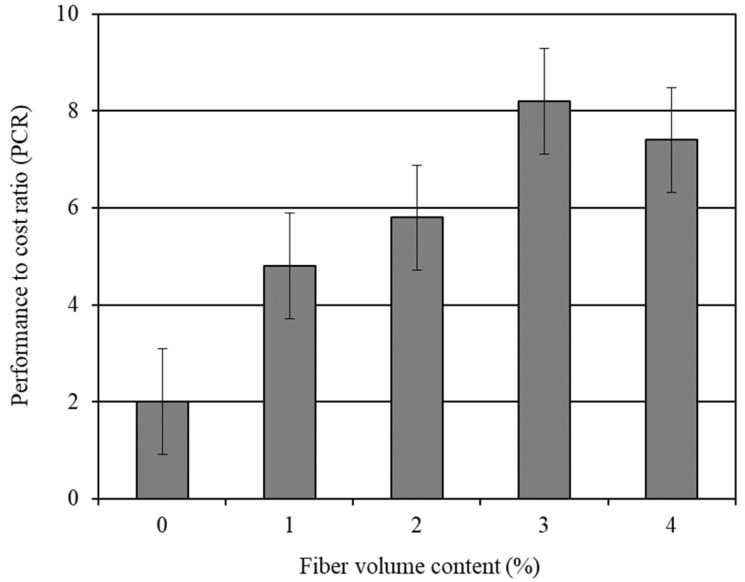
Influence of carbon fibers on the performance to cost ratio of mortar composite.

The effect of the carbon fiber volume content on the PCR of CFRM is graphically presented in Figure 5. It can be seen from Figure 5 that the PCR increased for the CFRM composites, which included up to a 3% carbon fiber volume content. Beyond the 3% carbon fiber content, the PCR of CFRM started to drop. The PCR of CFRM3 (the mortar composite with 3% carbon fibers) was slightly more than four times the PCR of OPCM (the mortar with 0% carbon fibers).

The workability, strength, impact resistance, and durability properties are extremely vital for any cement composite if it is intended to provide a high performance during its service life. At the same time, the cost of the cement composite is equally important. The optimum benefits are always desirable at a reasonable cost. In the context of the present study, CFRM3, which included 3% carbon fibers, offered the highest PCR (see Figure 5), and therefore is proven to be the most cost-effective mortar composite.

## 6. Optimum Mortar Composite

The optimum mortar composite was defined based on the overall performance regarding workability (flow time and volume flow), air content, unit weight, strength, and durability properties, as well as by considering the performance to cost ratios of various CFRMs. In general, the air content increased but the unit weight decreased when the carbon fibers were incorporated into the mortar composite. However, the increase in air content and the decrease in unit weight were the lowest for 3% carbon fibers. The inverted slump cone flow time, flexural strength, and impact resistance of CFRM composite increased, whereas its volume flow decreased with the higher volume content of the carbon fibers. Furthermore, the compressive strength increased for up to 3% carbon fibers, and thereafter it dropped significantly. Conversely, the water absorption decreased for up to 3% carbon fibers, and then it increased substantially for 4% carbon fibers. The overall test results revealed that the mortar composite CFRM3, which included 3% carbon fibers, possessed adequate workability for placing and compaction by vibration, maximum compressive strength, excellent flexural strength, and good impact resistance. Among all of the CFRMs, CFRM3 had the lowest air content and the highest unit weight. CFRM3 also exhibited the potential for good durability, as it had the lowest water absorption. Moreover, the highest performance to cost ratio was achieved for CFRM3. Hence, CFRM3 was the most cost-effective mortar composite. In summary, CFRM3 can be considered as the optimum mortar composite from all perspectives.

## 7. Conclusions

- The flow time of the CFRM composite increased significantly with the increasing volume content of carbon fibers, which decreased the workability of mortar because the water demand to wet the constituent materials increased due to the large surface area of the fibers. Consequently, the volume flow of CFRM significantly decreased with the increased amount of carbon fibers.
- The unit weight of CFRM was lower than that of OPCM because of the increased air content and light weight of the carbon fibers. The CFRM mix with 3% carbon fibers possessed the highest unit weight among all of the CFRM mixes, because of its relatively low air content and high packing condition of constituent materials.
- The air content of CFRM was greater than that of OPCM because of the more entrapped air voids. However, CFRM3 with 3% carbon fibers possessed the lowest air content among all of the CFRMs due to its adequate workability, which helped some of the air bubbles depart the mortar mix during vibration.
- The compressive strength of CFRM increased with the inclusion of carbon fibers up to a 3% volume content when the fresh composite was sufficiently workable and well compacted. Such an improvement in compressive strength was attributed to the good interfacial bond of carbon fibers with the surrounding mortar matrix.
- The flexural strength of CFRM increased with the increasing carbon fiber content due to the greater resistance to crack propagation and the improved bond strength in the presence of silica fume.
- The impact resistance of CFRM increased extensively with the greater volume content of carbon fibers due to increased anchorage and the enhanced interfacial bond between the fibers and mortar matrix in the presence of silica fume.
- The incorporation of carbon fibers diminished the water absorption of CFRM when the mortar composite possessed sufficient workability and received good compaction.
- The PCR of CFRM was substantially higher than that of OPCM. This suggests that CFRM can be cost-effective, prevailing over the high material cost of carbon fibers. The highest PCR was obtained for the CFRM including 3% carbon fibers.
- CFRM3 including 3% carbon fibers can be considered as the optimum mortar composite, because it provided adequate workability, maximum compressive strength, relatively high unit weight and low air content, excellent flexural strength, good impact resistance, the lowest water absorption, and the highest performance to cost ratio.

## Figures and Tables

**Table 1:** Properties of the constituent materials of the mortar composites.

Constituent Material	Property	Value or Percentage
River sand	Moisture content (wt. %)	0.50%
	Specific gravity (SSD *)	2.60
	Water absorption (wt. %)	1.60%
	Fineness modulus	1.97
Cement	Moisture content (wt. %)	0.50%
	Specific gravity	3.15
	pH	12–13
Silica fume	Moisture content (wt. %)	1.10%
	Specific gravity	2.20
Pitch-based carbon fibers	Ultimate tensile strength	1770 MPa
	Young’s modulus	180 GPa
	Specific gravity	1.85
	pH	7
Tap water	Turbidity	2.07 NTU
	pH	7
	Color	<5.00 TCU
	Total dissolved solids	18 mg/L
Naphthalene-based superplasticizer	Specific gravity	1.20
	Solid content (wt. %)	40%
	pH	8

* Saturated surface-dry.

**Table 2:** Mix proportions of the different mortar composites.

Type of Mortar Mix	Binder * (kg/m^3^)	Sand (kg/m^3^)	Carbon Fibers		Water (kg/m^3^)	Superplasticizer (kg/m^3^)
			(vol. %) ^†^	(kg/m^3^)		
OPCM	1124.6	556.2	0	0	394.1	11.3
CFRM1	1079.1	533.7	1	18.5	372.8	21.6
CFRM2	1056.5	522.4	2	37.0	359.8	31.7
CFRM3	1033.7	511.3	3	55.5	346.7	41.4
CFRM4	1011.1	500.0	4	74.0	334.1	50.6

* Cement plus silica fume (15% of binder), ^†^ Based on concrete volume.

**Table 3:** Properties of the different freshly mixed mortar composites.

Type of Mortar Mix	Inverted Slump Cone Flow		Unit Weight (kg/m^3^)	Air Content (%)
	Volume Flow (L/s)	Flow Time (s)		
OPCM	1.4	4	2070	1.0
CFRM1	1.0	5.5	1970	5.2
CFRM2	0.8	7	1945	6.4
CFRM3	0.6	8.5	1990	4.2
CFRM4	0.4	16.5	1905	7.9

**Table 4:** Properties of the different hardened mortar composites.

Type of Mortar Mix	Compressive Strength (MPa)	Flexural Strength (MPa)	Impact Resistance (J)	Water Absorption (wt. %)
OPCM	39.0	5.8	94.1	13.8
CFRM1	49.8	8.0	645.6	13.5
CFRM2	56.7	9.0	961.6	12.7
CFRM3	63.0	9.9	1378.6	11.2
CFRM4	47.7	10.4	2810.9	15.6

**Table 5:** PCRs of the different mortar composites.

Type of Mortar Mix	Volume Flow Factor (*V_ff_ = V_fm_*/*V_fc_*)	Compressive Strength Factor (*C_sf_ = C_sm_*/*C_sc_*)	Flexural Strength Factor (*F_sf_ = F_sm_*/*F_sc_*)	Impact Resistance Factor (*I_rf_ = I_rm_*/*I_rc_*)	Water Absorption Resistance Factor (*W_arf_ = W_am_*/*W_ac_*)	Cost Factor (*C_f_ = C_m_*/*C_c_*)	PCR
OPCM	1	1	1	1	1	1	2
CFRM1	0.7143	1.2769	1.3793	6.8608	1.0222	2.7020	4.8
CFRM2	0.5714	1.4538	1.5517	10.2189	1.0866	4.4222	5.8
CFRM3	0.4286	1.6154	1.7069	14.6504	1.2321	6.1403	8.2
CFRM4	0.2857	1.2231	1.7931	29.8714	0.8846	7.8567	7.4

## Data Availability

The data underlying this article will be shared on reasonable request from the corresponding author.
